# The sweet and embellishing *Lycium arabicum* Schweinf. ex Boiss. fruit oil: a potential source of essential ω-6 and ω-9 fatty acids, phytosterols, and carotenoids

**DOI:** 10.55730/1300-0527.3488

**Published:** 2022-08-11

**Authors:** Jawhar FAKHFAKH, Marwa AFFES, Hazem JABEUR, Mohamed AYADI, Noureddine ALLOUCHE

**Affiliations:** 1 Laboratory of Organic Chemistry (LR17ES08) Natural Substances Section, Faculty of Sciences of Sfax, University of Sfax, Sfax, Tunisia; 2 National Olive Oil Office, Sfax, Tunisia; 3 Olive Tree Institute, Sfax, Tunisia

**Keywords:** *Lycium arabicum*, edible oil, essential fatty acids, carotenoids, sugars

## Abstract

In our current phytochemical investigation on the Tunisian plant *Lycium arabicum* Schweinf. ex Boiss., we attempted to explore the oil obtained from its fruits. This oil was extracted by cold maceration with ethanol and was analyzed to estimate its sterol and fatty acid content. The physicochemical parameters of the oil were also examined. They were specified in terms of acid value (0.8 ± 0.01 mg KOH g^−1^ oil), peroxide value (10.9 ± 0.02 meq O_2_ kg^−1^ oil), saponification value (162.97 ± 0.03 mg KOH g^−1^ oil), chlorophylls (1.011 ± 0.03 mg kg^−1^ oil) and carotenoids (42.1 ± 0.05mg kg^−1^ oil). Gas chromatography analysis demonstrated that oleic (33.5%), palmitic (26.81%) and linoleic (21.51%) acids were the dominant fatty acids. Furthermore, the analysis of this oil with HPLC equipped with a differential refractometer detector (DRD) showed that Palmitic-Oleic-Oleic (21.10%) and Oleic-Oleic-Oleic/Palmitoleic-Palmitic-Palmitic (17.11%) are the main triacylglycerols in this oil. It was also found that this oil contains high levels of ***β***-sitosterol (31.51%), ***Δ***-5-Avenasterol (25.17%), and ***Δ***-7-Avenasterol (15.22%). Analysis of its spectroscopic features allowed us to deduce that this oil contains some sugars like glucose, sucrose, and fructose as well as several carotenoid pigments. From this perspective, *Lycium arabicum* fruits oil (LAFO) maybe regarded as a worthwhile product that deserves supplementary consideration and investigation as a potentially new multi-purpose candidate for agro-food as a sweetener and a beautifier, in addition to its extreme usefulness for cosmetic and medicinal applications owing to its richness in phytosterols and conservative pigments.

## 1. Introduction

A wide variety of plants involves naturally vegetable oils. Every plant affords separate and distinctive oil. Yet, only a few of these plants are invested in economic significance. Vegetable oils play a primary role in human nutrition. They afford a high energy source and involve distinctive fatty acids, which are primordial for health and are not generated by the human body. The main source of vegetable oils lies in the seeds of annual plants and oil-bearing trees. At present, with the world’s growing demand for vegetable oils, oil-processing industries are seeking new alternative sources [1].

Oils and fats display multiple benefits and innovative uses, mainly for food and a variety of industrial purposes. They are investigated in creating cosmetic products and used in the painting domain that considers especially the oils having siccative properties. They are composed of a major fraction, which contains fatty acids and triacylglycerols, and an unsaponifiable fraction containing various bioactive substances such as sterols, terpenes, pigments, and other chemicals that are inert towards the saponification reaction. These minor compounds are considered characteristic of a given oil or fat [2, 3].

*Lycium arabicum* is one of the basic traditional Tunisian medicinal plant species. Indeed, traditional healers use the dry powder of the fruits and leaves were used to treat ophthalmic diseases [4]. Those from the shoots and flowers of the aerial parts were used as antidiabetic [5] and antihypertensive agents [6] while the leaves were used against intestinal worms [7]. The genus *Lycium* is distributed in temperate regions of the globe and the Mediterranean basin and has about 75 different species [8]. Four species represent the genus *Lycium* in Tunisia. These are *L. arabicum* Boiss., *L. intricatum* Boiss., *L. europaeum* L., and *L. halimifolium* Mill. [9]. *L. arabicum* is a member of the Solanaceae family. It is a thorny shrub with multiple branches; the fruits are fleshy, with red berries when ripe. This tree can reach a height of 3 m. In Tunisia, it grows wild in the subhumid and semiarid bioclimatic areas.

Few studies reported in the literature and conducted on this plant showed that the latter contains diverse compounds such as sesquiterpene lactones, coumaric acid derivatives, flavonoids, sterols, anthraquinones [10] and flavonoid glucosides [11] while the biological investigation showed an interesting antioxidant and antimicrobial activities of fractions derived from the 80% ethanolic extract of fruits [12]. Other reports highlighted the hypoglycemic potential of this plant [13] as well as its antiplasmodial and antitrypanosomal activities [14].

On the other hand, and in prior research, the authors of the current paper have addressed the phytochemical composition and the biological potential of this plant. They inferred that leaves and stems stand for a potent source of fatty acids, quinic acid derivatives, flavonoids and amide alkaloids. In addition, the methanol extracts proved to exhibit outstanding antioxidant and lipase inhibitory activities [15]. In another work, the same authors described a polysaccharide from the fruits of this plant called LAP. They examined the chemical and physicochemical features of this polysaccharide and they found that it is endowed with potent antioxidant potential. Besides, they found that this polysaccharide exerts a remarkable protective impact upon human erythrocytes [16]. Furthermore, the essential oil of this plant was studied and constituted the focal point of a recently submitted paper for publication.

*L. arabicum* fruits exhibit numerous benefits for human health. However, in Tunisia, *L. arabicum* is a scarcely exploited species despite its high potential as a rich source with high nutritional and economic value. Deeper and more thorough knowledge about its biological activities and chemical composition would pave the way for the investment of this natural resource as a source of phytochemicals as well as a tool for economic and agronomic progress [15].

To the best of the authors’ knowledge, although the merits of *L. arabicum* fruits oil were patented, there is no research work in the literature concerning its physicochemical properties, fatty acids, triacylglycerols, and sterols constitution. The chief purpose of the current work is to specify for the first time the physicochemical features, fatty acids, sterols contents, and spectroscopic features of *L. arabicum* fruits oil (LAFO), which serve as a supporting argument for the possible use of this oil as an edible one.

## 2. Materials and methods

### 2.1. Plant material

Fruits of *L. arabium* were gathered (January 2020) from old trees growing wild in Sfax (Tunisia) from the region located at (34°56′25″ N and 10°54′31″ E). The taxonomic identification of the plant material was proven by Professor Mohamed Chaieb from the botany laboratory at the Faculty of Sciences, Sfax University, Tunisia. The voucher specimen (N° LCSN118) was deposited at the Laboratory of Organic Chemistry (Natural Substances section) of the same Faculty.

### 2.2. Oil extraction

Eight hundred g of air-dried fruits of *L. arabicum* was ground to fine particles using a domestic miller and then macerated three times with 2 L of ethanol for 24 h at room temperature using a mechanical stirrer. The obtained mixture was filtered through a filter paper (Whatman no.4) and the solvent was evaporated under reduced pressure at 40 °C giving 58.5 g of orange-colored oil stored at −20 °C until subsequent analysis.

### 2.3. Determination of physicochemical parameters

Acid value, saponification value, and peroxide value were calculated based on the corresponding standards [17], [18], and [19], respectively.

#### 2.3.1 Refractive index

A Sopelem series 3269 refractometer (Sopelem, France) was used to measure the refractive index at 25 °C.

#### 2.3.2 Spectroscopic indices (K232, K270), UV-Vis spectrum

The standard procedure [20] was used to measure the spectroscopic indices, K232 and K270 utilizing a UV-Vis spectrometer (Camspe M550 Double Beam Scanning UV/Vis). The latter was used for recording the UV-Vis spectrum of the oil too.

#### 2.3.3. Unsaponifiable matter determination

The unsaponifiable matter was specified referring to the standard [21].

#### 2.3.4. Viscosity determination

Oil viscosity was measured using a rheometer (Thermo electron Corporation, Haake Roto Visco 1). A computer software program (Rheowin 3.14 Job Manager) was used for data processing. All measurements were carried out at 25 °C with the use of a steel cone plate (C40/4) at a constant shear rate of 100 s^−1^.

#### 2.3.5. Density measurement

An Anton Paar (DMA 500) Density meter was used for the measurement of the density at 20 °C.

#### 2.3.6. Color measurement

The CieLab coordinates (L*, a*, b*) were specified using a chromameter (Conica Minolta CR-400/410) equipped with a data processor DP-400. The calibration of the apparatus was performed using a white title furnished with the device. In this color scale, L* denotes the sample’s lightness (100 = white, 0 = black), a* indicates redness when positive and greenness when negative, and b* corresponds to yellowness when positive and blueness when negative.

### 2.4. Chemical analysis of the oil

#### 2.4.1. Carotenoid and chlorophyll contents

The procedures reported by Minguez-Mosquera [22] were used for the assessment of the contents of carotenoid and chlorophyll pigments. Indeed, 7.5 g of LAFO was poured into a falcon tube and solubilized in 25 mL of cyclohexane. The chlorophyll part was recorded at (630, 670, and 710 nm) while the carotenoids part was specified at 470 nm using a UV-Vis spectrophotometer (Jenway 6320D Spectrophotometer). The pigment’s concentration was calculated in terms of:


eq. 1 $$(Chlorophylls\;mg/kg)=\frac{Abs670-(Abs630+Abs710)/2}{0.1086\times L}$$

eq. 2 $$\left(Carotenoids\;mg/kg)=(Abs470\times 10^{3}\times 25)/(2000\times 7.5\right)$$

In the Eq. (1), L denotes the thickness of the UV-Vis spectrophotometer cell (1 cm), and 0.1086 indicates a variable coefficient associated with the spectrometer.

#### 2.4.2. Fatty acid composition

The fatty acid composition was specified by alkaline treatment of *L. arabicum* fruits oil (LAFO) sample through supplementing 0.05 g of oil mixed with 1 mL of n-hexane by 1 mL of KOH solution (2 N) in methanol yielding a mixture of fatty acid methyl esters (FAMEs). These FAMEs were subjected to gas chromatography analyses through the use of a Shimadzu 17 A gas chromatograph equipped with a flame ionization detector (FID) and a capillary column operating as follows: the injector temperature was established at 230 °C, the column temperature at 180 °C, and the detector at 250 °C. The used carrier gas was nitrogen with a head pressure set at 0.6 bars. The used pressure of the air for the FID was 1.5 bar and that of hydrogen was 0.8 bar. For chromatographic separation, 1 μL of the mixture was injected onto a capillary column (TG225MS; ref: 26083-1430) having the trades below: polar stationary phase (cyanopropylmethyl/phenylmethyl-polysiloxane, 1:1, v/v), diameter 0.32 mm, length 30 m and film thickness 0.25 μm. The flow rate of the carrier gas was 30 mL s^−1^ and the split ratio was (40:1). Identifying the peaks was achieved by comparing the retention times to those of authentic compounds provided by Sigma-Aldrich. The fatty acid composition was determined after the addition of an internal standard invested in normalizing the chromatographic peak areas and was calculated in terms of relative percentages of each identified fatty acid.

#### 2.4.3. Triacylglycerol composition

The triacylglycerol composition of LAFO was determined following the International Olive Oil Council standard [23]. A solution of oil (5% w/v) was prepared in acetone in a graduated flask. A reverse-phase HPLC (HP 1100, Agilent Technology) apparatus was invested to carry out the triacylglycerols (TAGs) profile. This apparatus was equipped with a differential refractometer detector (DRD) and fitted with a C18 column (diameter 4.6 mm, length 250 mm, particle size 5 μm) using a mixture of acetonitrile/acetone (50:50, v/v) as eluent. Twenty μL of the sample was injected, and the analysis was carried out at a flow rate of 1.5 mL/min for 70 min. Comparing the retention time of the obtained peaks with those of authentic TAGs permitted the identification of the fruit oil’s TAGs. The composition in TAGs of the analyzed oil was presented as a relative percentage of each TAG. Moreover, equivalent carbon number (ECN) was used to allow the assignment of compounds for which no analytical standards were available.

ECN was computed according to the equation ECN = CN–2DB, where CN stands for the number of carbon atoms and DB corresponds to the number of double bonds [24].

#### 2.4.4. Sterol analysis

LAFO unsaponifiable matter was obtained and specified as instructed by the International Olive Oil Council [25]. First, 1 g of oil was refluxed for 1 h using 10 mL of an ethanolic solution of potassium hydroxide (10%, w/v). A few antibumping granules were added to the mixture. Then, 10 mL of distilled water was supplemented with the obtained solution. Next, it was extracted three times with 20 mL portions of ether to separate the unsaponifiable matter. After recombining, the ether extracts were dried over anhydrous sodium sulphate, filtered, and then evaporated *in vacuo* at 40 °C in a rotary evaporator. The obtained unsaponifiable matter was afterwards subjected to TLC separation using a mixture of (n-hexane/diethyl ether, 65:35, v/v) as a solvent system. After completing the development, the sterol band was monitored under 254 nm, scraped from the plate, and then extracted by methylene chloride. The gained sterols were silylated using the silylant mixture (pyridine–hexamethyldisilazane–trimethylchlorosilane, 9:3:1, v/v/v) amounting to 50 μL for every mg of sterols. The obtained trimethylsilyl ether sterols’ derivatives were analyzed on an Agilent Technologies (7890 A) GC apparatus equipped with an HP-5MS column (30 m × 0.25 mm i.d) (19091s-433UILTM) by injecting a volume of 1 μL. The detector and the injector temperatures were set at 320 °C, whereas the column was heated at 150 °C for 2 min. The temperature rose to 180 °C within 5 min, then to 270 °C within 2 min, and then to 300 °C within 15 min. Helium was used as carrier gas.

#### 2.4.5. NMR method

NMR spectra were measured on a Bruker Ascend 400 NMR Spectrometer functioning at 400 MHz for ^1^H and 100MHz for ^13^C nuclei using typical experiments. Thirty mg of LAFO was pooled in an NMR tube after being dissolved in 2 mL CDCl_3_. On the other hand 500 mg of LAFO was submitted to liquid-liquid extraction using water-chloroform as a solvent system. The water layer rich in sugar was separated, lyophilized, dissolved in 2 mL D_2_O and then loaded onto an NMR tube. Coupling constants (*J*) are denoted in Hertz and the chemical shifts (*δ*) in ppm [24].

#### 2.4.6. Infrared analysis

The FTIR spectra of LAFO were recorded on a Perkin Elmer Spectrum 100 FT-IR spectrometer equipped with a deuterated triglycerine sulphate (DTGS) detector. Sixty-four scans were invested under the resolution of 4 cm^−1^. The data interval given by the apparatus to get a resolution of 4 cm^−1^ was 1 cm^−1^. Duplicate spectra were registered for the same sample. All spectra were recorded in the range of 400–4000 cm^−1^ and handled with the computer software program spectrum (Perkin Elmer spectrum version 10.03.09).

### 2.5. Statistical analysis

Analyses were entirely conducted in triplicate, and data were displayed as means ± standard deviation (SD).

## 3. Results and discussion

### 3.1. Physicochemical properties of L. arabicum fruits oil

The *L.arabicum* fruits oil (LAFO) is liquid with dark orange color at room temperature. Table 1 displays the comparison of the physicochemical parameters of LAFO with those of olive oil [26]. The fruits of *L. arabicum* provided 7.3% of oil. Compared to the oils obtained from oleaginous plants, this value is low. This low yield could be explained by the fact that *Lycium* is not an oleaginous plant, although edible.

As far as the authors know, no literature data have tackled the physicochemical parameters for oil extracted from other *Lycium* species. As far as we are concerned, our focal point is upon comparing the parameters of LAFO with those of Chemlali olive oil (COO) [26]. This oil is commonly edible in Tunisia and even in multiple other countries since Tunisia exports a large quantity of this oil to other countries around the world.

Acid value can be used to determine the quality of the oil. It reflects either the free fatty acids amount present in oil or the hydrolysis degree of the oil. In addition, it indicates the edibility and suitability of oil for industrial uses. The acid value of LAFO (0.8 ± 0.01 mg KOH g^−1^ oil) proved to be comparable with that of COO (0.33 ± 0.00 mg KOH g^−1^ oil), which indicates that LAFO is edible [27]. Furthermore, the saponification value (162.97 ± 0.03 mg KOH g^−1^ oil) proved to be lower than that of COO (190.03 ± 0.08 mg KOH g^−1^ oil). The low saponification value indicates that the number of ester bonds is low and the mean molecular weight of fatty acids is high.

The peroxide value of LAFO (10.9 ± 0.02 meq O_2_ kg^−1^ oil) is comparable to that of COO (13.81 ± 3.53 meq O_2_ kg^−1^ oil). This peroxide value refers basically to the presence of high amounts of unsaturated fatty acids such as oleic, linoleic, and linolenic acids. Since this value is lower than 20 meq O_2_ kg^−1^ oil, this oil can be stored for a long time with a small deterioration [28].

The stability against oxidation stands as one of the key parameters to be taken into account in the industrial application of edible fruit seed oil. Seed oil oxidative stability, defined as the resistance to oxidation in process and during storage, may be evaluated using certain oxidation indices such as K232 and K270. K232 consisted of the specific extinction coefficient at 232 nm. It is an indicator of the primary oxidation degree of the oil and is related to the quantity of hydroperoxides species [29, 30]. LAFO exhibited a low absorptivity at 232 nm (1.37 ± 0.01), which is lower than that of COO (3.00 ± 0.01). Thus, it displayed a few quantities of hydroperoxides. On the other side, LAFO contains little amount of secondary oxidation compounds (α-diketones and α-unsaturated ketones) as its K270 is low (0.14 ± 0.02) [31].

When oil undergoes a deterioration by photooxidation, its pigments like chlorophylls and carotenoids act, oppositely. Indeed, chlorophylls initiate, with the presence of oxygen and light energy, photosensitized oxidation of unsaturated fatty acids and esters, inducing the deterioration of oil [32, 33]. Contrarily, carotenoids play the role of light filters through quenching singlet oxygen, which protects edible oil from photooxidation deterioration [34, 35].

LAFO contains a small number of green pigments, mainly chlorophylls, basically quantified by measuring absorbance at 630, 670, and 710 nm [22]. Chlorophyll tenor was estimated as (1.011 ± 0.03 mg kg^−1^), which is significantly lower than that of COO (11.30 ± 1.56 mg kg^−1^) (Table 1). Yet, the carotenoid content in LAFO specified in terms of (42.1 ± 0.05 mg kg^−1^) is responsible for the orange color and is significantly higher than that of COO. Accordingly, aiming to quantify this color, LAFO was subjected to color measurements by measuring the CieLab (L*, a*, b*) coordinates of LAFO. These coordinates were determined as L* = 30.67 ± 0.67, which is less than many other edible oils. The L* value explains the low brightness and the matt appearance of this oil. However, the positive values of a* (11. 89 ± 0.10) and b* (12.78 ± 0.87) stand for a good indicator of the red and yellow colors respectively, providing the orange color coming from the carotenoid pigments. The orange color is highly needed by the oil and fat industry, especially as it simulates the butter appearance and prevents the use of primary colorants like apocarotenals, carotenes, and annattos [36].

### 3.2. Fatty acid composition

The fatty acid contents of LAFO compared to that of other species of *Lycium* were depicted in Table 2. The main three fatty acids included in decreasing order of abundance are oleic C18:1 (33.5% ± 0.12%), palmitic C16:0 (26.81% ± 0.07%), and linoleic C18:2 (21.51% ± 0.11%) acids which together constitute around 81.82% of the total fatty acids of the oil. This order in the fatty acid composition of LAFO is different from those reported by Boulila and Bejaoui for *L. ruthenicum* [37] as well as Skenderidis et al. for *L. barbarum* and *L. chinense* [38]. Indeed, Linoleic (54.11% ± 0.10%, 37.89% ± 0.1%, 40.71% ± 0.0%), oleic (20.70% ± 0.07%, 20.07% ± 0.1%, 19.29% ± 0.0%), and palmitic (12.49% ± 0.03%, 18.96% ± 0.0%, 21.59% ± 0.0%) acids were the most frequent ones from the latter species of *Lycium* respectively. The total unsaturated fatty acid (TUFA) of LAFO was 69.13%. This value is higher than that of *L. barbarum* (54.49%) but lower than those of *L. ruthenicum* and *L. chinense* giving 80.57% and 70.18% of TUFAs respectively. This high amount of TUFA is very important in terms of human disease prevention and body nutrition. Oleic and linoleic acids have positive effects on human health especially owing to their preventive role in cardiovascular diseases and their eradicating effect on the serum LDL cholesterol. In addition, they are highly significant in nervous cell construction since they are not produced by human organism [39, 40]. Oleic and linoleic acids also possess many pharmacological traits such as antioxidant, anticancer, hypolipidemic, antiaggregating, antithrombosis, and antiglycemic activities [41, 42].

The investigated oil was marked by a polyunsaturated/saturated (P/S) ratio of 0.96, comparable to that of olive oil (0.92). This value is indicative that it is favorable in preventing heart diseases [43] and mitigating the systolic blood pressure in the cardiovascular system.

### 3.3. Triacylglycerol composition

**Figure 1:**
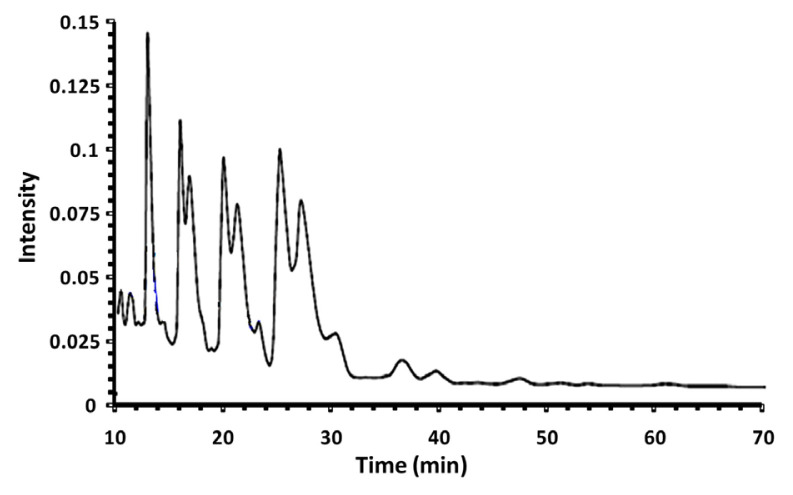
HPLC-DRD chromatographic profile of *L. arabicum* fruits oil (LAFO).

Reversed-phase HPLC equipped with a differential refractometer detector (DRD) was used to analyze the *L. arabicum* triacylglycerols (TAGs) (HPLC-DRD chromatographic profile in Figure 1). Identifying the major peaks was undertaken through the use of a standard TAGs mixture.

Table 3 summarizes the distribution of TAGs in LAFO along with their equivalent carbon number (ECN = CN–2DB; CN is the carbon number of the considered TAG and DB is the double bond’s number). The obtained results revealed that this oil contains 31 triacylglycerols with equivalent carbon number (ECN) ranging from 42 to 52.

The main LAFO TAGs were POO (21.10%) and OOO/PoPP (17.11%), followed by SLL/PLO (12.92%), OOLn/PoOL (10.82%), OOL/LnPP (10.67%) and OLL (8.53%). These predominant TAG species accounted for 81.15% of the total existing TAGs. Other minor TAGs components are presented in the same Table. Therefore, these data are consistent with the fatty acid profile showing oleic and palmitic acids as the major FAs of this oil with respective proportions of 33.5% and 26.8%. The TAG composition of LAFO proved to be different from that of the oil extracted from another *Lycium* species: *L. europaeum* [44], as depicted in Table 3. Indeed, it was found that this oil exhibited LLL (39.87%) and OLL/PLL (39.43%) as the main TAGs and the ECN ranges from 36 to 48. These differences could be due to the variability of the studied species. The oil’s TAG profile could be used to characterize the studied species.

### 3.4. Sterol composition

Phytosterols correspond to the most representative compounds of the oil’s unsaponifiable fraction. They are widely analyzed for tracking commercial frauds [45]. Previous studies reported that phytosterols play an intrinsic role in lowering lipid and serum cholesterol levels in humans [46]. Furthermore, they act as antiatherogenicity, antioxidant, anticancer, and antiinflammatory agents [41, 42]. Thus, much interest has been devoted to identify the phytosterols of fruit oil to make correlations with their medicinal and nutritional values. The main sterols of this oil (Table 4) are *β*-sitosterol (31.51% ± 0.76%) followed by *Δ*-5-avenasterol (25.17% ± 1.4%), *Δ*-7-avenasterol (15.22% ± 0.26%), stigmasterol (7.17% ± 0.11%) and cholesterol (6.24% ± 0.13%). This sterol composition is comparable to those reported in previous papers for *Lycium barbarum* and *Lycium chinense* oils with little differences [38]. *β*-sitosterol is the most frequent sterol in edible vegetable oils.

Triterpenic alcohols (erythrodiol and uvaol) constitute a part of the unsaponifiable oil fraction, and are explored along with the sterol fraction [47]. On the other side, it was evidenced that the triterpenic alcohols (erythrodiol and uvaol) play an important antioxidant role against lipid peroxidation in vitro and also reduce the production of hydrogen peroxide by stimulating macrophages in a dose-dependent way [48, 49]. Furthermore, Allouche et al. argued that uvaol and erythrodiol sharply decrease thrombin formation [48]. These compounds have many benefits for human health and are used to treat some cardiovascular diseases.

### 3.5. Spectroscopic analyses

#### 3.5.1 UV/Vis spectrophotometric analyses

**Figure 2:**
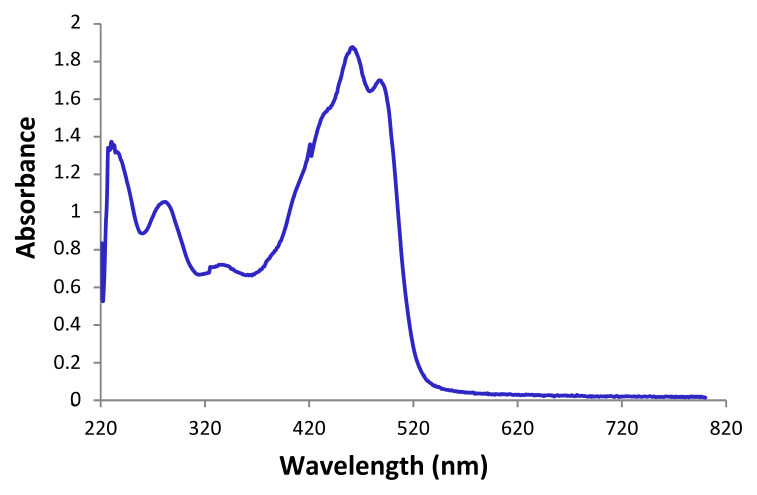
UV/Vis spectrum of *L. arabicum* fruits oil (LAFO) in CH_2_Cl_2_.

*L. arabicum* oil displayed absorbances in the UV-A(290–320 nm), UV-B(320–400 nm)and UV-C (400–600 nm) ranges (Figure 2). In the UV-B and the UV-A ranges, the wavelengths of ultraviolet light beget the most cellular damage [50]. Thus, LAFO could be utilized in several cosmetic preparations like UV skin protectors.

Similar properties were also deduced for *L. barbarum* oil [51], which proved to be a good protector against UV light owing to its richness in carotenoids.

LAFO UV spectrum exhibited a large and intense absorption band at around 430 nm contrary to many other edible oils like linseed or olive oils [24]. This specific band corroborates the presence of the high amount of carotenoids recorded previously in terms of (42.1 ± 0.05 mg kg^−1^). Carotenoids are powerful antioxidant substances having an intrinsic role in the reactions of neutralization of free radicals (FR) (notably reactive oxygen species ROS). In the human body, carotenoid molecules exist in the tissue and can neutralize certain attacks of FR, especially ROS, before they are undermined. Human skin contains carotenoids, like α-, γ-, β-carotene, lutein, zeaxanthin, lycopene, and their isomers, which protect the living cells against oxidation [52]. Applying a lotion rich in carotenoids can intensely improve skin protection against UV lights. It is worth noting that zeaxanthin dipalmitate is the major carotenoid in the fruits of different species of the genus *Lycium*, which stands for about one-half of the total carotenoid content [53].

#### 3.5.2 Fourier-transform infrared (FT-IR) characterization

**Figure 3:**
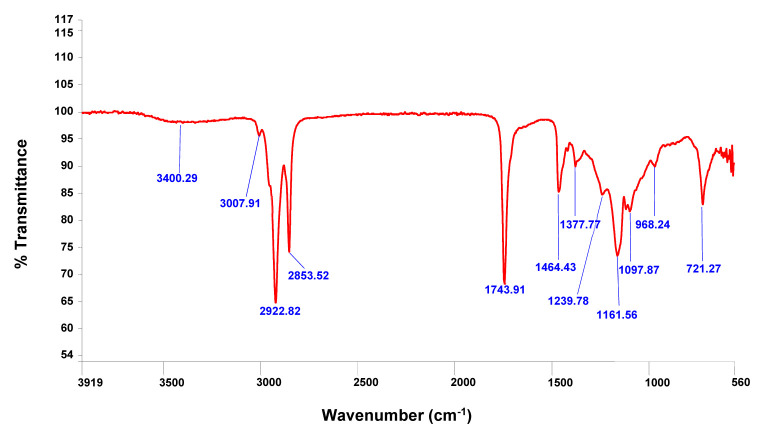
FT-IR spectrum of *L. arabicum* fruits oil (LAFO).

The FT-IR spectrum of *L. arbicum* fruit oil obtained at optimum conditions has been used to characterize this edible oil. This spectrum is plotted in Figure 3 and showed a typical bearing of a mixture of TGs. The broad bands in the 2923–2853 cm^−1^ region are attributable to C–H symmetric and asymmetric stretching vibrations of aliphatic CH_2_ and CH_3_ groups. The sharp, intense peak at 1743.9 cm^−1^ is related to (–C=O) carbonyl groups of triglyceride molecules and free fatty acids. The band situated at 1464 cm^−1^ is due to the CH_2_ and CH_3_ bending vibrations. That at 1377 cm^−1^ is related to the CH bending vibrations of *cis*-olefinic and those at 1239, 1097, and 1161 cm^−1^ correspond to the C–O and C–C stretching vibrations [24, 54]. The absorption bands of the other minor compounds like sterols, sugars, and carotenoids were observed quite weak and/or overlapped by those of the TGs, thus it was hardly depicted from this spectrum.

#### 3.5.3 NMR analyses

**Figure 4:**
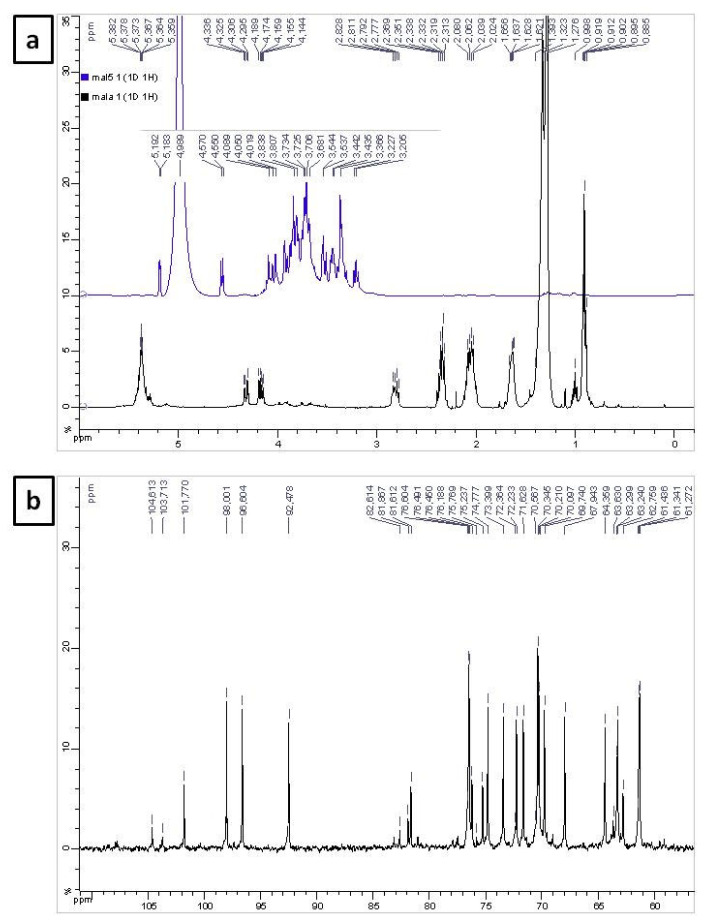
(a): ^1^H-NMR spectra of *L. arabicum* fruits oil (LAFO) (in black) and rich sugar fraction (in blue) performed in CDCl_3_ for LAFO and D_2_O for the sugars’ fraction at 400MHz (b): ^13^C-NMR spectrum of the rich sugar fraction in D_2_O at 100MHz.

The ^1^H-NMR spectrum of LAFO presented in Figure 4 reveals characteristic peaks of triacylglycerol moieties. In addition, it embeds weak signals in the region at *δ*_H_ 3.0–5.0, which are generally characteristics of sugar. To verify this hypothesis, we attempted to separate the water-soluble portion by conducting a liquid-liquid separation of the oil using water-chloroform as a solvent system. The water layer was collected, lyophilized, and checked again by NMR. Figure 4a confirms the presence of sugars. Indeed, it demonstrates two characteristic doublets at *δ*_H_ 5.19 (d, *J* = 3.7 Hz) and *d*_H_ 4.56 (d, *J* = 7.9 Hz) for *a-* and *b-* configuration of anomeric protons in glucose, respectively [55]. The oxy-methine and oxy-methylene protons of the sugar rings appear as signals between *δ*_H_ 3.20 and 4.08. The complexity of this spectrum allows deducing that other sugar moieties are present with glucose in this fraction. The presence of sugars corroborates the complexity of its ^13^C NMR spectrum (Figure 4b), showing six anomeric carbon signals between *δ*_C_ 92.4 and 104.6, among which the peaks at *δ*_C_ 92.4 and 96.6 are due to *a-* and *b-*configuration of anomeric carbons of glucose, respectively [56]. The peaks at *δ*_C_ 98.0 and 104.6 are probably assigned to sucrose and the other peaks at *δ*_C_ 101.7 and *δ*_C_ 103.7 are attributed to fructose [55]. The remaining peaks between *δ*_C_ 61.2 and 64.3 refer to the oxy-methylene protons and those between *δ*_C_ 67.9 and 83.1 are related to the oxy-methine protons of sugars.

The intense signals of the native LAFO ^1^H-NMR spectrum (Figure 4a) are representative of triacylglycerol species and appear as a triplet at *δ*_H_ 0.90 (t, *J* = 7.0 Hz) related to terminal methyl groups, intense multiplets at *δ*_H_ 1.27 and 1.32 owing to the methylene protons of the hydrocarbon chain, a multiplet centered at *δ*_H_ 1.63 assigned to *β*-methylene protons to carbonyl, a multiplet emerging at *δ*_H_ 2.06 related to methylene protons of allylic groups, two triplets centered at *δ*_H_ 2.332 and 2.338 (t, *J* = 7.6 Hz) referring to *α*-methylenes to carbonyl groups, two triplets at *δ*_H_ 2.79 and 2.81 (t, *J* = 6.2 Hz) characterizing diallylmethylene protons, two doublet of doublets centered at *δ*_H_ 4.16 (dd, *J_1_*= 12.0 and *J_2_* = 6.0 Hz) and *δ*_H_ 4.31 (*J_1_* = 12.0 and *J_2_* = 4.3 Hz) related to glyceryl-methylenes, a multiplet at *δ*_H_ 5.28 overlapped with another one at *δ*_H_ 5.36 related to olefinic protons and glyceryl-methines respectively [24]. These data were in good accordance with the iodine value and the fatty acid composition of the oil.

The oil (LAFO) addressed in this research work was extracted from *L. arabicum* fruits and was characterized in this manuscript for the first time. This oil is edible since the whole fruit is edible too. LAFO was obtained with a low yield as compared to other plants like olive, linseed, etc., which are oleagenic plants that provide a high yield of oil to around 30% depending on the species, the location, the harvest date, and many other parameters.

Meanwhile, this oil presented all the required characteristics of edible oil starting from the acid value, which is a very crucial parameter to figure out the quality of an oil. This parameter recorded in terms of 0.8 ± 0.01 mg KOH/g oil proved to be in the accepted range and indicated the low amount of free fatty acids, and therefore, its high stability. Saponification value, peroxide number, and K232 and K270 were comparable with those of olive oil, which further confirms the edibility of this oil. While chlorophylls were present in a small amount, carotenoids, which largely participate in the conservation of the oil through their antioxidant action, existed in a high amount compared to olive oil. The low concentrations of chlorophylls and high concentrations of carotenoids were expected as it was quite obvious that the fruit has an orange color. Measuring the CieLab coordinates enabled the quantification of this feature. Likewise, the UV-Vis spectrum displayed a broad, intense band at around 420 nm, originating basically from the carotenoid pigments. These pigments were also detected by FTIR analysis through the characteristic absorbance bands at 1161, 1464 and 3007 cm^−1^ [57]. The sugar content of LAFO was first detected by ^1^H-NMR spectroscopy, and then further extracted from LAFO, analyzed again, and identified as a mixture of glucose, sucrose, and fructose. In this way, the UV spectrum showed little absorbance of sugars due to their low absorbtivity in these wavelengths. The presence of these sugars which is not negligible, is presumably responsible for the low viscosity (57.05 ± 0.13 mPa.s) compared to that of olive oil (84.54 **±** 0.24mPa.s), albeit the densities of these oils were comparable (0.9346 ± 0.0053 g cm^−3^ for LAFO *versus* 0.9160 **±** 0.0047 g cm^−3^ for olive oil).

This oil can be regarded as a new and potential supplier of essential fatty acids ω-3, ω-6 and ω-9. In addition, it proved to contain remarkable amounts of sugars and carotenoids, which make this oil sweet and orange in color. On the other side, owing to its richness in carotenoids, this oil can be added to other edible oils like olive, soja, and linseed oils to support them against oxidation and/or endow an orange color making them more attractive and appealing to the consumer.

## 4. Conclusions

In the present study, thorough physicochemical and chemical features of fruit oil of *L. arabicum* (LAFO) growing wild in Tunisia were assessed and compared to other *Lycium* species. *L. arabicum* exhibited a few discrepancies in physicochemical and chemical traits with other *Lycium* species from other countries. Overall, the physicochemical parameters of LAFO indicated that this oil is edible, can be stored for a long time with small deterioration and is stable against oxidation. This study showed that this oil is rich in fatty acids (FAs) especially linoleic (21.5 ± 0.11%) and oleic (33.5 ± 0.12%) acids usually called ω-6 and ω-9 respectively. These FAs are essential for human health. Since human body is not able to synthesize them, they should be provided from out-of-body sources. HPLC analysis of this oil revealed that it contains 31 triacylglycerols (TAGs) (ECN42 to ECN52) of which POO (21.10%) and OOO/PoPP (17.11%) were the main ones. Investigation of the unsaponifiable matter revealed that *β*-sitosterol (31.51% ± 0.76%), *Δ*-5-avenasterol (25.17% ± 1.4%) and *Δ*-7-avenasterol (15.22% ± 0.26%) were the major phytosterols of this fraction. They play a crucial role in lowering lipid and serum cholesterol levels in human bodies. Spectroscopic investigation of this oil showed that it contains an important amount of sugars especially glucose, fructose and saccharose which make it sweet. Further, spectroscopic and chemical examination of this oil showed that it contains a high concentration of carotenoids (42.1 ± 0.05 mg kg^−1^) which not only protects this oil from photooxidation deterioration but also gives a very appealing and nice orange appearance. Therefore, it can be used as embellishing oil when added to other commercial foods like edible oils or butter for attracting consumers.

## Figures and Tables

**Table 1:** Comparison of the physicochemical properties of *L. arabicum* fruits oil (LAFO) with Chemlali olive oil.

Parameters	LAFO	Chemlali olive oil [26]
Yield (%)	7.31	14.34 ± 0.52
Acid value (mg KOH g^−1^ of oil)	0.80 ± 0.01	0.33 ± 0.00
Saponification number (mg KOH g^−1^ oil)	162.97 ± 0.03	190.03 ± 0.08
Peroxide number (meq O_2_ kg^−1^ oil)	10.9 ± 0.02	13.81 ± 3.53
K232	1. 37 ± 0.01	2.20 ± 0.00
K270	0.14 ± 0.02	0.17 ± 0.01
Carotenoids (mg kg^−1^)	42.1 ± 0.05	4.55 ± 0.40
Chlorophylls (mg kg^−1^)	1.011 ± 0.03	11.30 ± 1.56
Density (g cm^−3^ at 20 °C)	0.9346 ± 0.0053	0.9160 ± 0.0047^a^
Viscosity^c^ (mPa s)	57.05 ± 0.13	84.54 ± 0.24^a^

Values are mean **±** standard error of three replicates

c Viscosity was measured at γ**˙= 100 s**^−1^

a Result was measured in this study.

**Table 2:** Comparison of the fatty acid composition of *L. arabicum* fruits oil (LAFO) with *L. ruthenicum, L. barbarum*, and *L. chinense*.

Fatty acid	Carbon length	Fatty acid composition (%)			
		LAFO	*L. ruthenicum* [37]	*L. barbarum* [38]	*L. chinense* [38]
Saturated					
Myristic	14:0	0.22 ± 0.03	0.14 ± 0.01	-	-
Pentadecylic	15:0	0.07 ± 0.01	-	-	-
Palmitic	16:0	26.81 ± 0.07	12.49 ± 0.03	18.96 ± 0.0	21.59 ± 0.0
Margaric	17:0	0.01 ± 0.00	0.29 ± 0.02	-	-
Stearic	18:0	2.60 ± 0.03	3.56 ± 0.04	2.61 ± 0.0	1.78 ± 0.0
Arachidic	20:0	1.11 ± 0.05	2.48 ± 0.05	1.86 ± 0.0	1.14 ± 0.1
Behenic acid	22:0	*^ND^*	*^ND^*	3.95±0.2	0.56 ± 0.0
Lignoceric acid	24:0	*^ND^*	0.47± 0.04	6.03 ± 0.0	0.75 ±0.1
Monounsaturated					
Palmitoleic	16:1 (ω-7)	5.98 ± 0.13	0.76 ± 0.01	1.01 ± 0.0	1.66 ± 0.0
Margaroleic	17:1	0.05 ± 0.02	*^ND^*	*^ND^*	*^ND^*
Oleic	18:1 (ω-9)	33.50 ± 0.12	20.70 ± 0.07	20.07± 0.1	19.29± 0.0
Gondoic	20:1 (ω-9)	0.06 ± 0.02	*^ND^*	*^ND^*	*^ND^*
Polyunsatured					
Linoleic	18:2 (ω-6)	21.51 ± 0.11	54.11 ± 0.10	37.89 ± 0.1	40.71 ± 0.0
Linolenic	18:3 (ω-3)	8.03 ± 0.21	4.99 ±0.03	1.86 ± 0.0	8.52 ± 0.0
SAFA		30.82	19.43	33.41	25.82
MUFA		39.59	21.47	21.08	20.95
PUFA		29.54	59.10	39.75	49.23
P/S		0.96	3.04	1.19	1.90

*^ND^*: not detected; SAFA: saturated fatty acids; MUFA: monounsaturated fatty acids; PUFA: polyunsaturated fatty acids; P/S: (polyunsaturated/saturated) ratio

**Table 3:** Comparison of the triacylglycerol composition of *L. arabicum* fruits oil (LAFO) with *L. europaeum*.

Triacylglycerol	ECN	Composition (%)	
		LAFO	*L. europaeum* fruits oil [44]
LnLnLn	36	-	0.15
LnLLn	38	-	0.24
LLLn	40	-	3.58
LLL	42	5.74	39.87
OLLn+PoLL	42	0.12	-
PLLn	42	0.26	-
OLLn/LnLP	42	-	1.65
OLL	44	8.53	-
OLL/PLL	44	-	39.43
OOLn+PoOL	44	10.83	-
PLL+PoPoO	44	0.82	-
POLn+PPoPo+PPoL	44	0.11	-
OOL+LnPP	46	10.68	-
OOL/POL	46	-	9.41
PoOO	46	0.23	-
SLL+PLO	46	12.92	-
PoOP+SPoL+SOLn+SPoPo	46	0.52	-
PLP	46	2.14	-
OOO+PoPP	48	17.12	-
SOL	48	1.10	-
POO	48	21.10	-
POO/OOO	48	-	1.72
POP	48	4.41	-
SOO	50	2.33	-
POS+SLS	50	0.80	-
AOO	52	0.25	-

Ln: linolenic acid; L: linoleic acid; O: oleic acid; Po: palmitoleic acid; P: palmitic acid; S: stearic acid; A: arachidic acid; ECN: equivalent carbon number; (ECN = CN–2DB).

**Table 4:** Comparison of the sterol composition of *L. arabicum* fruits oil (LAFO) with *L. barbarum*.

Sterol	Composition (%)	
	*L. arabicum* (LAFO)	*L. barbarum* fruits oil [38]
Cholesterol	6.24 ± 0.13	5.2 ± 0.5
Ergosterol	-	13.8 ± 0.2
Brassicasterol	0.40 ± 0.05	-
24-Methylenecholesterol	0.42 ± 0.07	-
Campesterol	5.43 ± 0.14	-
Campestanol	0.46 ± 0.03	-
Stigmastrol	7.17 ± 0.11	11.6 ± 1.3
*β*-sitosterol	31.51 ± 0.76	39.4 ± 2.5
Sitostanol	2.15 ± 0.23	1.0 ± 0.1
*Δ*-5-Avenasterol	25.17 ± 1.4	14.5 ± 1.5
*Δ*-5-24-Stigmastadienol	3.37 ± 0.31	-
*Δ*-5-23-Stigmastadienol	-	12.6 ± 0.3
*Δ*-7-Stigmastenol	2.46 ± 0.17	0.8 ± 0.2
*Δ*-7-Avenasterol	15.22 ± 0.26	1.2 ± 0.3
Triterpenicalcohol di-hydroxyl		
Erythrodiol + Uvaol (%)	0.78 ± 0.06	-

## Abbreviations

LAFO: *Lycium arabicum* fruits oil
COO: Chemlali olive oil
FA: Fatty acid
FR: Free radicals
TAG: Triacylglycerols
SAFA: Saturated fatty acids
MUFA: Monounsaturated fatty acids
PUFA: Polyunsaturated fatty acids
TUFA: Total unsaturated fatty acids
P/S: polyunsaturated/saturated fatty acids
ECN: Equivalent carbon number
HPLC-DRD: High performance liquid chromatography equipped with a differential refractometer detector
UV: Ultraviolet
FT-IR: Fourier-transform infrared
NMR: Nuclear magnetic resonance
δ: Chemical shift, ppm
J: Coupling constant, Hz
A: arachidic acid
L: linoleic acid
Ln: linolenic acid
O: oleic acid
P: palmitic acid
Po: Palmitoleic acid
S: stearic acid
